# Optical Halo: A Proof of Concept for a New Broadband Microrheology Tool

**DOI:** 10.3390/mi15070889

**Published:** 2024-07-07

**Authors:** Jorge Ramírez, Graham M. Gibson, Manlio Tassieri

**Affiliations:** 1 Departamento de Ingeniería Química, Universidad Politécnica de Madrid, José Gutiérrez Abascal 2, 28006 Madrid, Spain; jorge.ramirez@upm.es; 2 School of Physics and Astronomy, Advanced Research Centre, University of Glasgow, Glasgow G11 6EW, UK; graham.gibson@glasgow.ac.uk; 3 Division of Biomedical Engineering, James Watt School of Engineering, Advanced Research Centre, University of Glasgow, Glasgow G11 6EW, UK

**Keywords:** rheology, microrheology, optical tweezers, complex fluids

## Abstract

Microrheology, the study of material flow at micron scales, has advanced significantly since Robert Brown’s discovery of Brownian motion in 1827. Mason and Weitz’s seminal work in 1995 established the foundation for microrheology techniques, enabling the measurement of viscoelastic properties of complex fluids using light-scattering particles. However, existing techniques face limitations in exploring very slow dynamics, crucial for understanding biological systems. Here, we present a proof of concept for a novel microrheology technique called “*Optical Halo*”, which utilises a ring-shaped Bessel beam created by optical tweezers to overcome existing limitations. Through numerical simulations and theoretical analysis, we demonstrate the efficacy of the *Optical Halo* in probing viscoelastic properties across a wide frequency range, including low-frequency regimes inaccessible to conventional methods. This innovative approach holds promise for elucidating the mechanical behaviour of complex biological fluids.

## 1. Introduction

The field of microrheology (i.e., the study of flow of matter at a micron length scale) has historical roots that go back to the pioneering work of the Scottish botanist Robert Brown, who first described the thermally driven random motion of pollen particles on the surface of water in 1827, a phenomenon that later has been referred to as “Brownian motion”. Since then, scientists of the calibre of Albert Einstein and Jean Perrin have contributed to establishment of a theoretical framework [1] explaining Brown’s observations and used this to corroborate their theories on “the discontinuous structure of matter” [2].

However, it was only in 1995 when Mason and Weitz published their seminal work [3] that the field of microrheology was established. They introduced a novel experimental method for obtaining the frequency-dependent linear viscoelastic (LVE) properties of complex fluids by measuring the time-dependent intensity autocorrelation function of the light scattered by microscopic spherical particles suspended into the fluids. In particular, they explicated the relationship between the trajectory of a probe particle undergoing Brownian motion acting as a motile scattering centre in their experiments and the fluids’ shear complex modulus governing the probe dynamics. Mason and Weitz’s work [3] has been the spark that has led to a variety of experimental and theoretical studies [4,5,6,7,8] that have limned the field of microrheology in two major areas: “*active*” and “*passive*” microrheology techniques. These two areas differ from each other depending on whether the particles’ motion is induced by an external force field such as in the case of magnetic tweezers (MT) [9,10], atomic force microscopy (AFM) [11,12], and optical tweezers (OT) [13,14,15,16,17,18,19,20,21] or it is generated by the thermal fluctuations of the fluid molecules surrounding the probe particles such as in the case of passive video particle tracking microrheology (PVPTM) [22,23], diffusing wave spectroscopy (DWS) [24,25] and dynamic light scattering (DLS) [26,27]. Notably, these techniques have revealed to be exceptionally sensitive transducers able to resolve forces as low as a few piconewtons and displacements as small as a few nanometers, with temporal resolutions of below a microsecond and sample volumes of a few microliters or less. Therefore, they have been promptly adopted by scientists for monitoring the dynamics of a variety of biological processes occurring at time and length scales commonly unaccessible to conventional bulk rheology methods, such as inside a living cell [28,29]. However, it must be said that microrheology measurements face significant challenges in non-homogeneous samples, as they assume uniform and homogeneous materials with linear viscoelastic properties. In reality, many relevant fluids are heterogeneous on a micrometer scale, leading to unreliable bulk properties due to varying local environments experienced by different probe particles. This undermines the continuum mechanical approach, making it ineffective for such materials. Therefore, while microrheology can be useful for small volumes of homogeneous fluids, classical mechanical rheometry is more suitable for larger samples, providing reliable information with less effort. Thus, our method is best applied to uniformly homogeneous samples with limited volume.

Notwithstanding their continuous development and successful applications throughout the *natural sciences*, there exists an ‘experimental limitation’ common to all of the existing microrheology techniques that prevents scientists to explore the very slow dynamics (i.e., occurring at frequencies <<0.01 Hz) of important biological and bio-analytical systems, including highly concentrated solutions of semiflexible (bio-)polymers (e.g., actin filaments [30,31,32,33,34,35] and/or DNA nano-tubes [36,37]). This is because there exists a threshold lag time ($\tau_{tr}$) in passive microrheology techniques above which measurements will not provide any valuable information about the rheological properties of the fluid under investigation. Indeed, for each of these techniques, $\tau_{tr}$ can be defined as the characteristic time taken by the probe particles to diffuse through (i) the focal plane of the microscope objective used to perform PVPTM measurements (which commonly has a thickness of circa 0.5 mm); (ii) the laser beam of circa 1 mm in diameter in the case of DLS; and (iii) a distance $l^{*}≃1$ mm in the case of DWS measurements. Interestingly, for the above-mentioned techniques, $\tau_{tr}$ rarely exceeds the 100 s and it can be estimated to a first approximation as $\tau_{tr}=L^{2}/D$, where *L* is the characteristic length of the microrheology techniques mentioned above and $D=k_{B}T/\left(6\pi a\eta_{0}\right)$ is the diffusion coefficient of the particles, which have a radius *a* and they are suspended into a fluid characterised by a zero shear viscosity $\eta_{0}$. $k_{B}$ is the Boltzmann constant and *T* is the absolute temperature. In the case of active microrheology techniques, they have a similar low frequency limit dictated mainly by the ‘patience’ of the operator (when of course materials’ related issues such as ageing, evaporation, and mutational effects [38] have been already pondered), as they all would require lengthy oscillatory measurements to access the materials’ low-frequency viscoelastic properties. In particular, these measurements would take a minimum duration time of 1.5×2π/ω seconds at best to return a reading of the materials’ viscoelastic properties at each explored frequency ω. It follows that, in order to explore the materials’ response within a frequency window ranging from ω=0.001 rad/s to ω=0.01 rad/s, at five frequency values distributed logarithmically within such window, the measurement would last 6 h and 40 min at the earliest.

Hence, the aim of this paper to present a theoretical framework and a schematic configuration of a novel microrheology technique based on optical tweezers, capable of exploring the fluids’ viscoelastic properties over the widest range of experimentally accessible frequencies, which is only limited at the top end by the acquisition rate of the device used for tracking the particle position, thus removing the aforementioned low frequency limits. This is possible by shaping the laser beam of a conventional optical tweezers rig from a single Gaussian beam into a ring-shaped Bessel beam by means of the combined action of a (diffractive) axicon lens and an ultra-fine spatial light modulator (SLM), as described in the following sections. The newly generated optical trap will have a toroidal shape (hence the name “*Optical Halo*”) with different stiffnesses depending on the direction to which the particle’s trajectory is projected onto. Notably, in contrast to conventional optical tweezers, where the ability to monitor the 3D motion of the trapped particle for an ‘infinite’ time is rendered ineffective for gathering low-frequency rheological information due to the nature of the trap itself (as its compliance overshadows the fluids’ viscoelastic properties at frequency values lower than the system’s “corner frequency” [39]), the *Optical Halo* offers a unique opportunity to monitor the ‘free diffusion’ of a probe particle along the azimuthal direction of the toroid for an ‘indefinite’ amount of time wherein, owing to trap geometry, the net restoring optical force is null. Thus, we enable scientists, for the first time ever, to perform broadband passive microrheology measurements on complex fluids characterised by very long relaxation times.

To validate the efficacy of the proposed microrheology method, we conducted numerical simulations of the thermally driven motion of a micron-sized probe particle confined by a ring-trap and immersed in both Newtonian and viscoelastic fluids. This involved numerically solving a generalised Langevin equation, which incorporates a time-dependent damping force modelled using a constitutive equation based on the Jeffreys model of a complex fluid while also considering non-symmetric trap stiffness.

## 2. Theoretical Background

### 2.1. Linear Rheology

The linear viscoelastic properties of a generic material can be expressed in terms of its shear complex modulus, $G^{*}\left(\omega \right)=G^{\prime }\left(\omega \right)+iG^{\prime \prime }\left(\omega \right)$, which is a complex number whose real and imaginary parts provide information on the elastic and the viscous nature of the material under investigation [40]. These are commonly indicated as the storage ($G^{\prime }\left(\omega \right)$) and loss ($G^{\prime \prime }\left(\omega \right)$) moduli. The conventional method for measuring the LVE properties of a material is based on the imposition of an oscillatory shear stress $\sigma \left(\omega ,t\right)=\sigma_{0}\sin\left(\omega t\right)$ (where $\sigma_{0}$ is the amplitude of the stress function) and the measurement of the resulting oscillatory shear strain, which would have a form such as $\gamma \left(\omega ,t\right)=\gamma_{0}\left(\omega \right)\sin\left(\omega t-\varphi \left(\omega \right)\right)$, where $\gamma_{0}\left(\omega \right)$ and φ(ω) are the frequency-dependent strain amplitude and phase shift between the stress and the strain, respectively. The relationship between the shear complex modulus and the two experimental functions describing the stress and the strain is as follows [40]:

(1) $$G^{*}\left(\omega \right)=\frac{\hat{\sigma }\left(\omega \right)}{\hat{\gamma }\left(\omega \right)},$$

where $\hat{\sigma }\left(\omega \right)$ and $\hat{\gamma }\left(\omega \right)$ are the Fourier transforms of σ(ω,t) and γ(ω,t), respectively. Notice that Equation (1) is of general validity, i.e., it applies to any temporal forms of the stress and the strain. In the particular case of sinusoidal functions, Equation (1) returns the following:

(2) $$\begin{array}{c} G^{*}\left(\omega \right)=\frac{\sigma_{0}}{\gamma_{0}\left(\omega \right)}\cos\left(\varphi \left(\omega \right)\right)+i\frac{\sigma_{0}}{\gamma_{0}\left(\omega \right)}\sin\left(\varphi \left(\omega \right)\right)\equiv G^{\prime }\left(\omega \right)+iG^{\prime \prime }\left(\omega \right), \end{array}$$

which provides the expressions of the moduli as function of both the frequency-dependent functions $\gamma_{0}\left(\omega \right)$ and φ(ω). Notice that $G^{*}\left(\omega \right)$ is time-invariant [40].

Over the past century, the frequency behaviour of the viscoelastic moduli has been correlated, both theoretically and experimentally [40,41,42,43], to the material’s topological structure and molecular interactions at different length scales, i.e., from the bulk sample at relatively low frequencies, down to atomic length scales for frequencies of the order of THz [44,45]. Hence, it is important to obtain knowledge over the widest possible range of frequencies to gain a full picture of the materials’ structure and dynamics.

### 2.2. Passive Microrheology

In their pioneering work, Mason and Weitz [3] demonstrated that when a micron-sized spherical particle is suspended into a fluid at thermal equilibrium, the analysis of its trajectory can provide information on the viscoelastic properties of the suspending fluid. In particular, they showed that the trajectory $\vec{r}\left(t\right)\;\forall \;t$ of a suspended bead is directly related to the LVE properties of the surrounding complex fluid by means of a generalised Langevin equation:

(3) $$m\vec{a}\left(t\right)={\vec{f}}_{R}\left(t\right)-\int_{0}^{t}\zeta \left(t-\tau \right)\vec{v}\left(\tau \right)d\tau$$

where *m* is the mass of the particle, $\vec{a}\left(t\right)$ is its acceleration, $\vec{v}\left(t\right)$ its velocity, and ${\vec{f}}_{R}\left(t\right)$ is the usual Gaussian white noise term, modelling stochastic thermal forces acting on the particle. The integral term, which incorporates a generalised time-dependent memory function ζ(t), represents viscous damping by the fluid. Using the assumption that the Laplace-transformed bulk viscosity of the fluid $\tilde{\eta }\left(s\right)$ is proportional to the microscopic memory function $\tilde{\zeta }\left(s\right)=6\pi a\tilde{\eta }\left(s\right)$, where *a* is the bead radius, they provided the solution to Equation (3) in terms of the particles’ mean squared displacement (MSD):

(4) $$G^{*}\left(\omega \right)={\left.s\tilde{\eta }\left(s\right)\right|}_{s=i\omega }=\frac{1}{6\pi a}\left[\frac{6k_{B}T}{i\omega \langle \Delta \hat{r^{2}}\left(\omega \right)\rangle }+m\omega^{2}\right]$$

where $k_{B}$ is Boltzmann’s constant, *T* is the absolute temperature, and $\langle \Delta \hat{r^{2}}\left(\omega \right)\rangle$ is the Fourier transform of the MSD, $\langle \Delta r^{2}\left(\tau \right)\rangle \equiv \langle {\left[\vec{r}\left(t+\tau \right)-\vec{r}\left(t\right)\right]}^{2}\rangle$. The average $\langle \ldots \rangle$ is taken over all initial times *t* and all particles if more than one is observed.

### 2.3. Range of Accessible Dynamics and Measurable Viscosities

As described in the introduction, despite continuous development and successful applications throughout the *natural sciences*, all existing microrheology techniques share an experimental limitation that prevents scientists from exploring the very slow dynamics of important biological and bio-analytical systems. These limitations also affect the range of measurable viscosities, especially at the top end, where significantly longer measurements are needed in the case of passive microrheology. In this regard, the range of viscosities that can be explored by microrheology measurements is dictated by the spatial and temporal resolutions of the experimental system and the ‘patience’ of the operator. Indeed, let us consider a Deborah number given by the following:

(5) $$De=\frac{\tau_{D}}{\tau_{ob}}=\frac{6\pi \eta ad^{2}}{\tau_{ob}k_{B}T}$$

This ratio compares a characteristic diffusion time $\tau_{D}$ (i.e., the time it takes for a particle of radius *a* to traverse a generic distance *d* in a fluid of viscosity η at thermodynamic equilibrium) to the observation time $\tau_{ob}$, defined as the lag time between frames describing the particle’s motion. Note that the lower limit of $\tau_{ob}$ is constrained by the inverse of the acquisition rate ($f_{AR}$) of the device used to detect the particle position, while the upper limit is determined by whichever fails first: the operator’s patience or the equipment’s data storage capacity. Therefore, in general by assuming that the spatial resolution of the experimental system is on the order of a few nanometres (i.e., $d_{sr}^{2}\approx 10^{-17}$ nm^2^) and that a typical trace particle has a radius on the order of microns, the following statements are true: (i) For lag times shorter than the characteristic diffusion time $\tau_{Dsr}$ (i.e., De>1) required by the tracer particle to diffuse a distance equal to $d_{sr}$ (or equivalently $\forall \tau_{ob}\leq \eta /20$ when considering the above figures and room temperature), the tracer particle will appear stationary to the observer, and the viscoelastic properties of fluids will result higher than their actual values. For example, in the case of water at room temperature (whose viscosity is $\eta ≅10^{-3}$ Pa·s) these artefacts will appear at acquisition rates of the order of 20 kHz or higher. (ii) For lag times longer than $\tau_{Dsr}$ (i.e., De<1 or equivalently $\forall \tau_{ob}\geq \eta /20$ under similar experimental conditions as above), long-duration measurements will be essential to determine the rheological properties of fluids characterised by a relatively high viscosity value. The new optical tweezer-based technique proposed in this article provides a robust solution for studying the viscoelastic properties of complex fluids across a broad frequency range, addressing the limitations of current microrheology methods as detailed below.

### 2.4. The Link between Bulk and Micro-Rheology

Let us retrieve a straightforward relationship between the thermally driven MSD of a probe particle and the time-dependent shear compliance J(t) of the suspending fluid. In conventional bulk rheology, the shear compliance is defined as the ratio of the time-dependent shear strain γ(t) to the magnitude $\sigma_{0}$ of the constant shear stress that is switched on at time t=0:

(6) $$J\left(t\right)=\gamma \left(t\right)/\sigma_{0}.$$

The compliance is related to the materials’ shear relaxation modulus G(t) by means of a convolution integral [40]:

(7) $$\int_{0}^{t}G\left(\tau \right)\;J\left(t-\tau \right)\;d\tau =t.$$

Moreover, given that the complex shear modulus $G^{*}\left(\omega \right)$ is also defined as the Fourier transform of the time derivative of G(t), by taking the Fourier transform of Equation (7) one obtains the following:

(8) $$G^{*}\left(\omega \right)=i\omega \hat{G}\left(\omega \right)=\frac{1}{i\omega \hat{J}\left(\omega \right)},$$

where $\hat{G}\left(\omega \right)$ and $\hat{J}\left(\omega \right)$ are the Fourier transforms of G(t) and J(t), respectively.

Furthermore, given that the Fourier transform is a linear operator, by equating Equations (4) and (8) one obtains the following:

(9) $$\langle \Delta \hat{r^{2}}\left(\omega \right)\rangle =\frac{k_{B}T}{\pi a}\hat{J}\left(\omega \right)\;\;\;⟺\;\;\;\langle \Delta r^{2}\left(\tau \right)\rangle =\frac{k_{B}T}{\pi a}J\left(t\right),$$

where it has been assumed that for micron-sized particles the inertial term $m\omega^{2}$ (otherwise present on the right side of Equation (4)) is negligible for frequencies much smaller than MHz and that J(0)=0 for viscoelastic fluids. Equation (9) expresses the linear relationship between the MSD of suspended spherical particles and the macroscopic creep compliance of the suspending fluid [46]. Therefore, it allows for the evaluation of the fluid’s complex shear modulus (via Equation (4)) without the need of any preconceived model once an effective analytical method for performing the Fourier transform of a discrete set of experimental data is adopted, like either of the two methods discussed in Ref. [47].

### 2.5. Hybrid Microrheology with Optical Tweezers

When the particle’s fluctuations are constrained by a stationary harmonic potential generated by optical tweezers, one could write a generalised Langevin equation similar to Equation (3), but with an additional term accounting for the trapping force:

(10) $$m\vec{a}\left(t\right)={\vec{f}}_{R}\left(t\right)-\int_{0}^{t}\zeta \left(t-\tau \right)\vec{v}\left(\tau \right)d\tau -\kappa \vec{r}\left(t\right),$$

where κ is the trap stiffness [48,49].

Following the same assumptions made by Mason and Weitz for the case of freely diffusing particles, Equation (10) can be solved for the materials’ complex modulus in terms of either the normalised mean squared displacement (NMSD) $\Pi \left(\tau \right)=\langle \Delta r^{2}\left(\tau \right)\rangle /2\langle r^{2}\rangle$ [17] or the normalised position autocorrelation function (NPAF) $A\left(\tau \right)=\langle \vec{r}\left(t\right)\vec{r}\left(t+\tau \right)\rangle /\langle r^{2}\rangle$ [18]:

(11) $$G^{*}\left(\omega \right)\frac{6\pi a}{\kappa }=\left(\frac{1}{i\omega \hat{\Pi }\left(\omega \right)}-1\right)\equiv {\left(\frac{1}{i\omega \hat{A}\left(\omega \right)}-1\right)}^{-1}\equiv \frac{\hat{A}\left(\omega \right)}{\hat{\Pi }\left(\omega \right)}$$

where $\hat{\Pi }\left(\omega \right)$ and $\hat{A}\left(\omega \right)$ are the Fourier transforms of Π(τ) and A(τ), respectively. The inertial term ($m\omega^{2}$) present in the original works [17,18] and in Equation (4) has been neglected here because for micron-sized particles it becomes significant only at frequencies of the order of MHz. For ‘sufficiently long’ measurements, it is straightforward to demonstrate that A(τ) and Π(τ) are simply related to each other [18]:

(12) $$\Pi \left(\tau \right)\equiv \frac{\langle r^{2}\left(t+\tau \right)\rangle +\langle r^{2}\left(t\right)\rangle -2\langle \vec{r}\left(t_{0}\right)\vec{r}\left(\tau \right)\rangle }{2\langle r^{2}\rangle }\equiv 1-A\left(\tau \right)$$

In addition, by Fourier transforming Equation (12), one obtains the following relation: $i\omega \hat{\Pi }\left(\omega \right)=1-i\omega \hat{A}\left(\omega \right)$; hence, we obtain the equivalences in Equation (11).

At this point, it is important to highlight that while Equation (10) holds general validity and is applicable to any viscoelastic material—whether liquid or solid—to determine its complex modulus without the need for any preconceived model to interpret the data, the same cannot be said for all microrheology studies adopting a Langevin equation with a time-invariant drag coefficient (i.e., 6πaη, where η is a constant viscosity). Therefore, to avoid potentially misleading information, the latter approach should be adopted *if and only if* the suspending fluid is Newtonian. Similar arguments apply when a Lorentzian function is used to fit the power spectral density (S(ω)) of the particle fluctuations—a procedure valid only for Newtonian fluids [39]. Furthermore, even when attempts are made to directly derive the viscoelastic properties of materials from the analysis of S(ω), the adoption of the error-prone Kramers–Kronig transformation algorithm may compromise the outcomes [47].

### 2.6. Jeffreys Model

**Figure 1:**
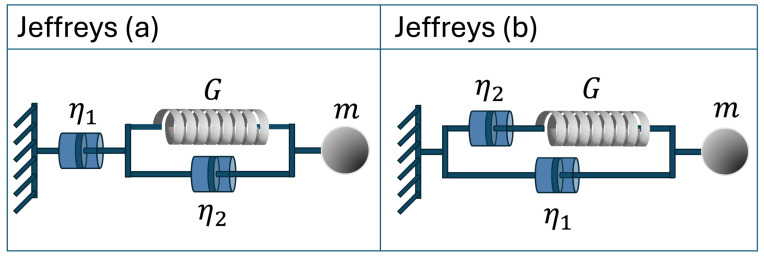
Two equivalent representations of Jeffreys model of a viscoelastic fluid: (**a**) is made of a dashpot (of viscosity $\eta_{1}$) connected in series with a Kelvin–Voight element, which is made of a dashpot (of viscosity $\eta_{2}$) and a spring (of modulus *G*) placed in parallel, whereas (**b**) is made of a dashpot (of viscosity $\eta_{1}$) connected i parallel with a Maxwell element, which is made of a dashpot (of viscosity $\eta_{2}$) and a spring (of modulus *G*) placed in series. Both models are connected to a material point of mass *m*, whose contribution to the dynamics of the system is neglected in this work.

There are many different mechanical models that aim to represent the rheological behaviour of viscoelastic materials. One such model is the Jeffreys model [50], which consists of an ensemble of simple mechanical elements such as springs and dashpots, combined in two different but equivalent configurations (see Figure 1). It must be noted that uniform linear viscoelastic fluids can often be more accurately described by more sophisticated multi-mode models. However, given that the aim of this paper is to present a proof of concept for a novel microrheology tool, we opted for the simpler Jeffreys model to keep the computational approach manageable and focused on demonstrating the core functionality of our new method without compromising its general effectiveness.

It is a straightforward exercise to derive the ordinary differential equations that describe the dynamic response of the two models shown in Figure 1. In particular, for the version of Jeffreys model shown in Figure 1a, the equation reads as follows:

(13) $$\eta_{1}\dot{\gamma }\left(t\right)+\frac{\eta_{1}\eta_{2}}{G}\ddot{\gamma }\left(t\right)=\sigma \left(t\right)+\frac{\eta_{2}+\eta_{1}}{G}\dot{\sigma }\left(t\right),$$

whereas for the version shown in Figure 1b, the constitutive equation is as follows:

(14) $$\sigma \left(t\right)+\frac{\eta_{2}}{G}\dot{\sigma }\left(t\right)=\left(\eta_{1}+\eta_{2}\right)\dot{\gamma }\left(t\right)+\frac{\eta_{2}\eta_{1}}{G}\ddot{\gamma }\left(t\right).$$

The two constitutive equations are very similar, and indeed, with careful selection of parameters in both models, one would obtain an identical dynamic response from both configurations. In what follows, we will focus on the version of the Jeffreys model shown in Figure 1b. However, for the sake of completeness, some additional derivations are provided in the SI for the version in Figure 1a. By Fourier transforming the constitutive Equation (14), the complex modulus of the model can be extracted (see details in the Supplementary Information):

(15) $$\frac{G^{*}\left(\omega \right)}{G}=\frac{1}{G}\frac{\hat{\sigma }\left(\omega \right)}{\hat{\gamma }\left(\omega \right)}=\frac{\omega^{2}\tau_{2}^{2}}{1+\omega^{2}\tau_{2}^{2}}+i\omega \left\{\tau_{1}+\frac{\tau_{2}}{1+\omega^{2}\tau_{2}^{2}}\right\},$$

where $\tau_{1}=\eta_{1}/G$ and $\tau_{2}=\eta_{2}/G$.

### 2.7. Generalized Langevin Equation for an Optically Trapped Particle Moving in a Jeffreys Medium

Let us now consider the case of an optically trapped particle suspended in a complex fluid whose rheological properties could be described by means of a Jeffreys model. The thermally driven fluctuation of the probe particle can be described by the following generalized Langevin equation:

(16) $$m\vec{a}\left(t\right)={\vec{f}}_{R}\left(t\right)-\zeta_{0}\vec{v}-\int_{0}^{t}\zeta_{p}\left(t-\tau \right)\vec{v}\left(\tau \right)d\tau -\kappa \vec{r}\left(t\right).$$

where $\zeta_{0}$ is the constant friction coefficient due to the Newtonian solvent (i.e., $\zeta_{0}=6\pi a\eta_{1}$ due to the dashpot in parallel in Jeffreys model in Figure 1b), $\zeta_{p}\left(t\right)$ is the memory kernel accounting for the temporary non-local drag force due to the viscoelastic nature of the complex fluid, which we will assume to have an exponential form (i.e., a Maxwell model, as the one depicted in parallel to $\eta_{1}$ in the Jeffreys model in Figure 1b). Interestingly, without the need for assumptions other than those underpinning the solutions of Equations (3) and (10), Equation (16) can be solved for the frequency-dependent viscoelastic properties of a Jeffreys fluid in terms of the Fourier transform of the particle normalised position autocorrelation function (see details in the Supplementary Information):

(17) $$\frac{\omega^{2}\tau \eta_{2}}{1+\omega^{2}\tau^{2}}+i\omega \left\{\eta_{1}+\frac{\eta_{2}}{1+\omega^{2}\tau^{2}}\right\}=\frac{\kappa }{6\pi a}\frac{i\omega \hat{A}\left(\omega \right)}{\left(1-i\omega \hat{A}\left(\omega \right)\right)},$$

where it has been assumed that $\zeta_{p}\left(t\right)$ has the following form:

(18) $$\zeta_{p}\left(t\right)=\frac{6\pi a\eta_{2}}{\tau }exp\left(-t/\tau \right)\;\;\;\forall t\geq 0,$$

whose Fourier transform is as follows:

(19) $${\hat{\zeta }}_{p}\left(\omega \right)=\frac{6\pi a\eta_{2}}{\left(1+i\omega \tau \right)}$$

Notably, the left side of Equation (17) is identical to the right side of Equation (15) when not scaled by *G*, which represents the dynamic response of the Jeffreys model shown in Figure 1b.

### 2.8. A Possible Experimental Configuration of an Optical Halo

Bowman et al. [51] discussed various methods for creating optical tweezers and their many applications. In the case of creating an annular optical trap, various options are available. Optical beam scanning, using a fast scanning focused laser, can be used to rapidly move between many trapping sites, such that the particles do not have time to diffuse away between visits by the laser [52]. Different methods of scanning can be used, such as galvo mirrors, or acousto-optic deflectors (AOD). However, such methods reduce the average exposure time and introduce a tangential drag force that may affect the tracking data. An alternative method is to create a fixed ring trap which does not require a laser to be scanned. One such method is based on diffractive optics and holography, and can be used to create arbitrary optical traps [53,54] (more commonly termed holographic optical tweezers (HOT)). In such systems a dynamic, computer controlled, diffractive optical element can be implemented using a liquid crystal spatial light modulator (SLM), and can be extended to allow the manipulation of particles in three dimensions. There are many examples of ring traps being created using SLMs [55,56]. However, these are often created using orbital angular momentum which prevents the particles from diffusing freely around the ring. SLM-based systems generally have a lower power efficiency which can limit the total power available in the ring trap and, more importantly, a high-resolution SLM is required to change the ring size and trapping depth. Even high-quality, efficient, SLMs can produce unwanted diffraction orders, leading to “Ghost Spots”, and steps must be taken to minimise these effects [57]. In contrast, an optical tweezers system based on an axicon, or conical lens, can provide a low-cost, efficient, and highly flexible solution [58,59].

**Figure 2:**
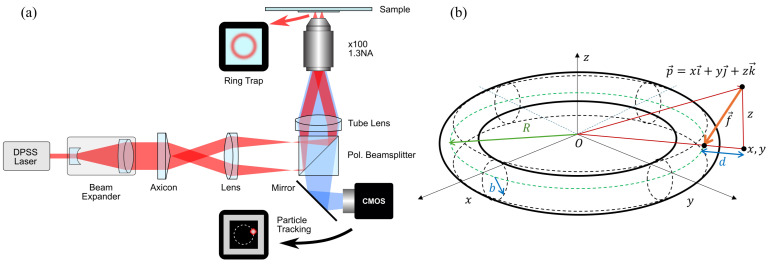
(**a**) A proposed experimental configuration of a ring-based optical trap. The setup is based on the configurations reported by Shao et al. [58], where an axicon is used to create a smooth optical ring. This can range from a simple fixed design to a design that allows the size of the ring traps to be adjusted [59]. (**b**) Geometrical representation of the torus-shaped optical halo. The torus has major radius *R* and minor radius *b* which are related to the stiffness of the halo in the radial direction by $\kappa_{r}=k_{B}T/b^{2}$. The stiffness of the optical halo in the *z* direction is $\kappa_{z}$.

A uniform annular trap requires that the incident light be composed of collimated beams from all directions (0–360 degrees azimuthal angle) all with the same angle to the optical axis, forming a cone of collimated light intersecting the back aperture of the objective lens. In order to fully utilise the numerical aperture of the objective lens, the thickness of the cone should be equal to the diameter of the back aperture of the objective. A proposed experimental configuration is depicted in Figure 2a. This design is compatible with compact optical tweezers systems, such as that reported by Gibson et al. [60], allowing a stable instrument to be developed.

### 2.9. Geometrical Representation of an Optical Halo

As shown in Figure 2b, the optical trap confines the motion of the particle to a three-dimensional toroidal (doughnut-shaped) region characterised by a major radius *R* and a circular cross-section of radius *b* (the minor radius). The toroid is centred at the origin of the coordinate system and revolves around the *z*-axis. Owing to the optical trap, a generic particle with coordinates $\vec{p}=x\vec{i}+y\vec{j}+z\vec{k}$ is attracted towards the generator circle with a force that is proportional to the minimal distance between the particle and the circle (orange arrow in Figure 2b). In this work, we consider the generic, and most realistic case, of an anisotropic toroidal trap characterised by two different stiffnesses $\kappa_{r}$ and $\kappa_{z}$ in the radial and axial directions, respectively. It follows that the force on a particle due to the toroidal optical trap can be expressed as follows:

(20) $${\vec{F}}_{OT}=-\kappa_{r}\left(1-\frac{R}{\sqrt{x^{2}+y^{2}}}\right)\left(x\vec{i}+y\vec{j}\right)-\kappa_{z}z\vec{k}$$

Notably, as we shall exploit hereafter, the 3D optically trapped particle will experience a net-zero force in the azimuthal direction of the toroid, i.e., $\kappa_{\theta }=0$. This unique characteristic of the *Optical Halo* trap enables the particle to undergo ‘one-dimensional free diffusion indefinitely’ while remaining within the microscope’s focal plane for optimal tracking.

### 2.10. Overdamped Particle Moving in a Newtonian Fluid with a Toroidal Optical Trap

The motion of a colloidal particle moving in a Newtonian fluid, discarding inertial effects, can be described by the following Brownian dynamics equations:

(21) $$\begin{array}{cc} dx= & -\frac{\kappa_{r}}{\zeta_{0}}\left(1-\frac{R}{\sqrt{x^{2}+y^{2}}}\right)xdt+\sqrt{\frac{2k_{B}T}{\zeta_{0}}}dW_{x}\\ dy= & -\frac{\kappa_{r}}{\zeta_{0}}\left(1-\frac{R}{\sqrt{x^{2}+y^{2}}}\right)ydt+\sqrt{\frac{2k_{B}T}{\zeta_{0}}}dW_{y}\\ dz= & -\frac{\kappa_{z}}{\zeta_{0}}zdt+\sqrt{\frac{2k_{B}T}{\zeta_{0}}}dW_{z} \end{array}$$

where $\kappa_{r}$ and $\kappa_{z}$ are the trap stiffness components in the radial and axial directions, respectively, and *R* is the major radius of the toroidal trap. $\zeta_{0}$ is the friction coefficient of the particle in the Newtonian fluid, which is defined by Stokes’ law $\zeta_{0}=6\pi a\eta_{0}$, where *a* is the radius of the particle and $\eta_{0}$ is the viscosity of the fluid. The variables $W_{\alpha }$, with α=x,y,z, are Wiener processes [61,62] with a mean of zero and variance equal to dt (the Wiener process represents the integral of a Gaussian white noise).

In the following, we use the square root of the variance of the particle position in the radial direction as unit of length and the inverse of the corner frequency $\zeta_{0}/\kappa_{r}$ as the unit of time to define the non-dimensional variables $\overset{ˇ}{r}$ and $\overset{ˇ}{t}$, respectively:

(22) $$r=\sqrt{\frac{k_{B}T}{\kappa_{r}}}\overset{ˇ}{r}\;\;\;\;\;t=\frac{\zeta_{0}}{\kappa_{r}}\overset{ˇ}{t}$$

It follows that the system of (21) can be written in its dimensionless form:

(23) $$\begin{array}{cc} d\overset{ˇ}{x}= & -\left(1-\frac{\overset{ˇ}{R}}{\sqrt{{\overset{ˇ}{x}}^{2}+{\overset{ˇ}{y}}^{2}}}\right)\overset{ˇ}{x}d\overset{ˇ}{t}+\sqrt{2}d{\overset{ˇ}{W}}_{x}\\ d\overset{ˇ}{y}= & -\left(1-\frac{\overset{ˇ}{R}}{\sqrt{{\overset{ˇ}{x}}^{2}+{\overset{ˇ}{y}}^{2}}}\right)\overset{ˇ}{y}d\overset{ˇ}{t}+\sqrt{2}d{\overset{ˇ}{W}}_{y}\\ d\overset{ˇ}{z}= & -\frac{\kappa_{z}}{\kappa_{r}}\overset{ˇ}{z}d\overset{ˇ}{t}+\sqrt{2}d{\overset{ˇ}{W}}_{z} \end{array}$$

Interestingly, the dynamics of the particle depend only on two parameters: (i) the ratio between the trap stiffness in the axial direction to that in the radial direction $\kappa_{z}/\kappa_{r}$ and (ii) the major radius of the torus in its dimensionless form $\overset{ˇ}{R}$. For the sake of clarity, here we provide some characteristic values of the above mentioned parameters explored in this work, which have been chosen to resemble those encountered in real experimental cases. A very common solvent in microrheology measurements is water, with viscosity η=0.001 Pa·s. The probe particles have typical dimensions of $a=10^{-6}$ m, and the major radius of the toroid is assumed to be $R=10^{-5}$ m. A typical value of the trap stiffness is $\kappa_{r}=10^{-6}$ N/m, and the ratio between the axial and radial stiffness is assumed to be $\kappa_{z}/\kappa_{r}\approx 0.33$. It follows that the units of length and time are $6.41\cdot 10^{-8}$ m and $1.88\cdot 10^{-2}$ s, respectively, whereas in non-dimensional units, the radius of the particle is $\overset{ˇ}{a}=15.6$, and the major radius of the torus is $\overset{ˇ}{R}=155.9$.

## 3. Results and Discussion

**Figure 3:**
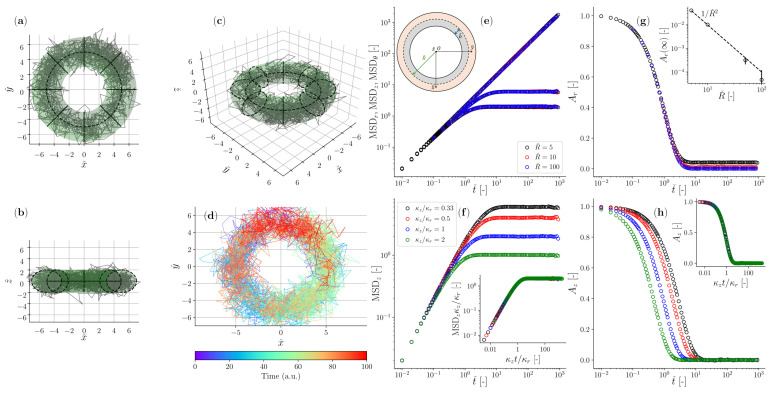
(**a**–**d**) Trajectory of a colloidal particle suspended in a Newtonian fluid and constrained by a toroidal optical trap with major radius $\overset{ˇ}{R}=5$, small radius $\overset{ˇ}{b}=1$, and $\kappa_{z}/\kappa_{r}=\frac{1}{3}$. (**e**–**h**) Similar simulation conditions to (**a**–**d**), but exploring the effects of varying $\overset{ˇ}{R}$ on the MSD in (**e**), and on the normalised position autocorrelation function in the radial $A_{r}\left(t\right)$ in (**g**). The inset in (**g**) shows the steady-state value of $A_{r}\left(t\right)$ as a function of $\overset{ˇ}{R}$. (**f**,**h**) show the effects of varying the ratio $\kappa_{z}/\kappa_{r}$ on the MSD in (**f**), and on the normalised position autocorrelation function $A_{z}\left(t\right)$, both only in the axial direction. The insets in (**f**,**h**) show the master curves for MSD_*z*_ and $A_{z}\left(t\right)$ when the same data shown in the main are plotted against $\kappa_{z}t/\kappa_{r}$, respectively, and the MSD_*z*_ is normalised by the variance of the optical trap in the *z* direction [26].

In Figure 3a–d, we report an example of a trajectory of a particle suspended in a Newtonian fluid drawn by means of Brownian dynamics simulations, demonstrating its confined diffusion within an *Optical Halo* trap of defined size and proprieties. In particular, the colour-coded Figure 3d illustrates that the particle can explore the entire volume defined by the toroidal trap when the measurement duration is sufficiently long. Hence, we have created an opportunity to conduct microrheology measurements at very low frequencies, which has never been achieved before. In Figure 3e, we report the mean squared displacements of the particle trajectory evaluated for all three independent components of the motion (i.e., radial *r*, axial *z*, and azimuthal θ directions) and for three different values of the major radius *R*. Notably, only the azimuthal component exhibits a linear mean squared displacement (MSD_*θ*_) proportional to *t*, akin to freely diffusing particles. Conversely, for both other components, the MSDs plateau at long times, as the constraining force of the optical trap outweighs the fluid’s compliance. Interestingly, none of the above MSDs reveal to be affected by the size of the torus, whose effect is actually apparent only when observing the time dependency of the position autocorrelation function in the radial direction $A_{r}\left(t\right)$, which does not converge to zero at long times for small values of the major radius *R*, as shown in Figure 3g. This can be understood by examining the slight difference in volume between the torus portion available on the inner side of the axial circle and the larger one on the outer side, as illustrated schematically in the inset of Figure 3e. Therefore, the particle has a higher probability to spend time at distances larger than the torus axis *R* (or equivalently, the particle position distribution along the radial direction has a negative skewness); thus, the position correlation function does not converge to 0 at large times (or equivalently, the normalised mean squared displacement does not converge to 1 because of Equation (12)). This is because the disparity between the inner ($A_{i}$) and the outer ($A_{o}$) areas (i.e., the grey and the orange areas of the inset of Figure 3e, respectively) of the planar cross-section of the toroid scales in terms of their ratio from $A_{i}/A_{o}=1/3$ for R=b, to $A_{i}/A_{o}=1$ for R→∞. Interestingly, the outcomes of the simulations inform us that this phenomenon vanishes as *R* increases (see inset of Figure 3g) with a scaling law of circa $A_{r}\left(\infty \right)\propto 1/R^{2}$.

The insets in (f) and (h) show the master curves for MSD_*z*_ and $A_{z}\left(t\right)$ when the same data shown in the main are plotted against $\kappa_{z}t/\kappa_{r}$, respectively, and the MSD_*z*_ is normalised by the variance of the optical trap in the *z* direction [26].

### 3.1. Overdamped Particle Moving in a Jeffreys Fluid with Toroidal Optical Trap

Let us now explore the dynamics of an optically trapped particle suspended in an ideal non-Newtonian medium such as a Jeffreys fluid. The motion of a colloidal particle moving in a single mode Jeffreys fluid, discarding inertial effects, can be described by a set of non-Markovian stochastic differential Equations (SDE) [63]. Introducing an additional stochastic variable, the system can be written as the following equivalent higher-dimensional Markovian SDE:

(24) $$\begin{array}{cc} dx= & -\left[\frac{\kappa_{r}}{\zeta_{0}}\left(1-\frac{R}{\sqrt{x^{2}+y^{2}}}\right)x+\frac{\kappa^{*}}{\zeta_{0}}Q_{x}\right]dt+\sqrt{\frac{2k_{B}T}{\zeta_{0}}\frac{\zeta_{0}}{\kappa^{*}\tau +\zeta_{0}}}dW_{x0}+\sqrt{\frac{2k_{B}T}{\zeta_{0}}\frac{\kappa^{*}\tau }{\kappa^{*}\tau +\zeta_{0}}}dW_{x1}\\ dy= & -\left[\frac{\kappa_{r}}{\zeta_{0}}\left(1-\frac{R}{\sqrt{x^{2}+y^{2}}}\right)y+\frac{\kappa^{*}}{\zeta_{0}}Q_{y}\right]dt+\sqrt{\frac{2k_{B}T}{\zeta_{0}}\frac{\zeta_{0}}{\kappa^{*}\tau +\zeta_{0}}}dW_{y0}+\sqrt{\frac{2k_{B}T}{\zeta_{0}}\frac{\kappa^{*}\tau }{\kappa^{*}\tau +\zeta_{0}}}dW_{y1}\\ dz= & -\left(\frac{\kappa_{z}}{\zeta_{0}}z+\frac{\kappa^{*}}{\zeta_{0}}Q_{z}\right)dt+\sqrt{\frac{2k_{B}T}{\zeta_{0}}\frac{\zeta_{0}}{\kappa^{*}\tau +\zeta_{0}}}dW_{z0}+\sqrt{\frac{2k_{B}T}{\zeta_{0}}\frac{\kappa^{*}\tau }{\kappa^{*}\tau +\zeta_{0}}}dW_{z1}\\ dQ_{x}= & -\left[\frac{\kappa_{r}}{\zeta_{0}}\left(1-\frac{R}{\sqrt{x^{2}+y^{2}}}\right)x+\frac{\kappa^{*}\tau +\zeta_{0}}{\zeta_{0}\tau }Q_{x}\right]dt+\sqrt{\frac{2K_{B}T}{\zeta_{0}}\frac{\kappa^{*}\tau +\zeta_{0}}{\kappa^{*}\tau }}dW_{x1}\\ dQ_{y}= & -\left[\frac{\kappa_{r}}{\zeta_{0}}\left(1-\frac{R}{\sqrt{x^{2}+y^{2}}}\right)y+\frac{\kappa^{*}\tau +\zeta_{0}}{\zeta_{0}\tau }Q_{y}\right]dt+\sqrt{\frac{2K_{B}T}{\zeta_{0}}\frac{\kappa^{*}\tau +\zeta_{0}}{\kappa^{*}\tau }}dW_{y1}\\ dQ_{z}= & -\left(\frac{\kappa_{z}}{\zeta_{0}}z+\frac{\kappa^{*}\tau +\zeta_{0}}{\zeta_{0}\tau }Q_{z}\right)dt+\sqrt{\frac{2K_{B}T}{\zeta_{0}}\frac{\kappa^{*}\tau +\zeta_{0}}{\kappa^{*}\tau }}dW_{z1} \end{array}$$

where most of the parameters have been already defined above and $\kappa^{*}$ is the effective stiffness of the Maxwell element in the Jeffreys model, with $\kappa^{*}=6\pi aG$, where *G* is the modulus of the elastic element of the Maxwell model, and τ is its characteristic relaxation time with $\tau =\eta_{2}/G$ (see Figure 1b). The friction coefficient $\zeta_{0}$ is now referred to the viscosity $\eta_{1}$ of the dashpot connected in parallel in Figure 1b. The auxiliary variables $Q_{x}$, $Q_{y}$, and $Q_{z}$ have units of length and allow to express the evolution as a set of Markovian stochastic differential Equations [63].

As in the case of Newtonian fluids, we use the square root of the variance of the position of the particle in the radial direction as unit of length for the positions *x*, *y*, and *z*, as well as for the auxiliary variable $Q_{x}$, $Q_{y}$, and $Q_{z}$, and the inverse of the corner frequency $\zeta_{0}/\kappa_{r}$ of the compound system (represented by the trap stiffness, the bead radius, and the purely viscous component of the Jeffreys model) as the unit of time to define the non-dimensional variables $\overset{ˇ}{r}$ and $\overset{ˇ}{Q}$ such that the following is true:

(25) $$r=\sqrt{\frac{k_{B}T}{\kappa_{r}}}\overset{ˇ}{r}\;\;\;\;\;t=\frac{\zeta_{0}}{\kappa_{r}}\overset{ˇ}{t}\;\;\;\;\;Q_{\alpha }=\sqrt{\frac{k_{B}T}{\kappa_{r}}}{\overset{ˇ}{Q}}_{\alpha }$$

It follows that the system of (24) can be written in its dimensionless form:

(26) $$\begin{array}{cc} d\overset{ˇ}{x}= & -\left[\left(1-\frac{\overset{ˇ}{R}}{\sqrt{{\overset{ˇ}{x}}^{2}+{\overset{ˇ}{y}}^{2}}}\right)\overset{ˇ}{x}+\frac{\kappa^{*}}{\kappa_{r}}{\overset{ˇ}{Q}}_{x}\right]d\overset{ˇ}{t}+\sqrt{\frac{2}{\mathrm{De}+1}}d{\overset{ˇ}{W}}_{x0}+\sqrt{\frac{2\mathrm{De}}{\mathrm{De}+1}}d{\overset{ˇ}{W}}_{x1}\\ d\overset{ˇ}{y}= & -\left[\left(1-\frac{\overset{ˇ}{R}}{\sqrt{{\overset{ˇ}{x}}^{2}+{\overset{ˇ}{y}}^{2}}}\right)\overset{ˇ}{y}+\frac{\kappa^{*}}{\kappa_{r}}{\overset{ˇ}{Q}}_{y}\right]d\overset{ˇ}{t}+\sqrt{\frac{2}{\mathrm{De}+1}}d{\overset{ˇ}{W}}_{y0}+\sqrt{\frac{2\mathrm{De}}{\mathrm{De}+1}}d{\overset{ˇ}{W}}_{y1}\\ d\overset{ˇ}{z}= & -\left(\frac{\kappa_{z}}{\kappa_{r}}\overset{ˇ}{z}+\frac{\kappa^{*}}{\kappa_{r}}{\overset{ˇ}{Q}}_{z}\right)d\overset{ˇ}{t}+\sqrt{\frac{2}{\mathrm{De}+1}}d{\overset{ˇ}{W}}_{z0}+\sqrt{\frac{2\mathrm{De}}{\mathrm{De}+1}}d{\overset{ˇ}{W}}_{z1}\\ d{\overset{ˇ}{Q}}_{x}= & -\left[\left(1-\frac{\overset{ˇ}{R}}{\sqrt{{\overset{ˇ}{x}}^{2}+{\overset{ˇ}{y}}^{2}}}\right)\overset{ˇ}{x}+\frac{\kappa^{*}}{\kappa_{r}}\frac{\mathrm{De}+1}{\mathrm{De}}{\overset{ˇ}{Q}}_{x}\right]d\overset{ˇ}{t}+\sqrt{2\frac{\mathrm{De}+1}{\mathrm{De}}}d{\overset{ˇ}{W}}_{x1}\\ d{\overset{ˇ}{Q}}_{y}= & -\left[\left(1-\frac{\overset{ˇ}{R}}{\sqrt{{\overset{ˇ}{x}}^{2}+{\overset{ˇ}{y}}^{2}}}\right)\overset{ˇ}{y}+\frac{\kappa^{*}}{\kappa_{r}}\frac{\mathrm{De}+1}{\mathrm{De}}{\overset{ˇ}{Q}}_{y}\right]d\overset{ˇ}{t}+\sqrt{2\frac{\mathrm{De}+1}{\mathrm{De}}}d{\overset{ˇ}{W}}_{y1}\\ d{\overset{ˇ}{Q}}_{z}= & -\left[\frac{\kappa_{z}}{\kappa_{r}}\overset{ˇ}{z}+\frac{\kappa^{*}}{\kappa_{r}}\frac{\mathrm{De}+1}{\mathrm{De}}{\overset{ˇ}{Q}}_{z}\right]d\overset{ˇ}{t}+\sqrt{2\frac{\mathrm{De}+1}{\mathrm{De}}}d{\overset{ˇ}{W}}_{z1} \end{array}$$

where we have introduced the following Deborah number:

(27) $$\mathrm{De}=\frac{\tau \kappa^{*}}{\zeta_{0}}$$

which represents the ratio between the characteristic time of the fluid τ and a characteristic observation time defined as the inverse of the corner frequency of a particle moving in a Newtonian fluid with viscosity $\eta_{1}$ subjected to a trap of stiffness $\kappa^{*}$. It is worth remembering that for classical microrheology measurements with optical tweezers, the compliance of the optical trap overshadows that of the fluid for lag times longer than the inverse of the corner frequency [64,65], which is the key issue this article aims to solve.

The solution of the system of (26) depends on four parameters: the Deborah number De, the major radius of the torus *R*, the ratio between the axial and the radial stiffnesses $\kappa_{z}/\kappa_{r}$, and the ratio between the elastic component of the fluid and that of the optical trap in the radial direction $\kappa^{*}/\kappa_{r}$. However, it should be noted that alternative non-dimensional groups could have been defined. For example, it is possible to compute a second Deborah number ${\mathrm{De}}_{2}=\mathrm{De}\cdot \kappa_{r}/\kappa^{*}=\tau \kappa_{r}/\zeta_{0}$, which corresponds to the ratio between the characteristic time of the fluid and the characteristic observation time when the fluid is Newtonian and the stiffness of the trap is $\kappa_{r}$.

**Figure 4:**
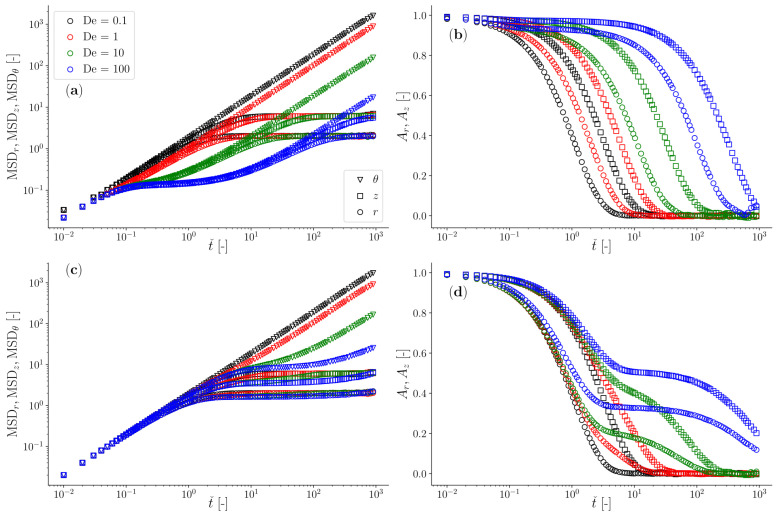
The mean squared displacement (**a**,**c**) and the normalized position autocorrelation function (**b**,**d**), evaluated along the three main directions *r*, *z*, and θ of a toroidal optical trap from Brownian dynamics simulations of an ensemble of 1000 particles on a torus with $\overset{ˇ}{R}=150$, a ratio of axial to radial stiffness of $\kappa_{z}/\kappa_{r}=1/3$; these are parametric (colour coded) using De, as depicted in the legend. The simulations were performed by assuming two different Jeffreys fluids: (i) a fluid with an elastic component stiffer than that of the optical tweezers, i.e., $\kappa^{*}/\kappa_{r}=16$ (**a**,**b**) and (ii) a fluid with an elastic component softer than that of the optical tweezers, i.e., $\kappa^{*}/\kappa_{r}=0.25$ (**c**,**d**). Note that the normalized position autocorrelation function for the azimuthal component is not reported in order to maintain clarity in the diagrams. This is because the data would consistently hover near a value of unity throughout almost the entire time window, falling to zero only at large lag times.

In Figure 4, we report the outcomes of Brownian dynamics simulations in terms of both the particle’s mean squared displacement and the normalised position autocorrelation function for two limiting cases: (i) when a viscoelastic fluid has an elastic component much bigger than the trap stiffness of the optical trap in the radial direction (being this one the strongest of the two constraining forces exerted by the *Optical Halo*), and (ii) *vice versa*, specifically with $\kappa^{*}/\kappa_{r}$ =16 and 0.25, respectively.

In the first case, the compliance of the viscoelastic fluid ($J\left(t\right)\propto 1/k^{*}$) is much lower than that of the optical trap ($J_{OT}\propto k_{r}^{-1}$)—or equivalently, $\kappa^{*}>>\kappa_{r}>\kappa_{z}>\kappa_{\theta }\equiv 0$—and the full spectrum of the materials’ linear viscoelastic properties can be retrieved from the analysis of either of the two time-averaged functions reported in Figure 4, even in the case of a classical instrument configuration with a single point optical trap. This is because the longest relaxation time of the fluid (here represented by De) is much shorter than the inverse of the corner frequency of the specific compound system (characterised by the fluid viscosity, bead radius, and trap stiffness [66]); therefore, the fluid compliance reaches its steady state (i.e., J∝t) well before being overshadowed by that of the optical trap (e.g., see data for De=100 in Figure 4a). In this case, the only requirement for performing broadband microrheology measurements is related to their duration ($T_{m}$), which for Newtonian fluids has been estimated to scale with the relative viscosity as $T_{m}\propto \eta_{r}^{3}$ [67], when an error of 1% is aimed. Notice that in the case of viscoelastic fluids, one should use the low-frequency (i.e., long-time) viscosity to estimate $\eta_{r}$.

In the second case, when the elastic component of the fluid is much weaker than that of the optical trap—or equivalently, $\kappa_{r}>\kappa_{z}>>\kappa^{*}>\kappa_{\theta }\equiv 0$—the determination of the viscoelastic properties of the fluid is hindered by the compliance of the optical trap both in the radial and axial directions, which start overshadowing the compliance of the complex fluid at much earlier lag times, often well before the longest relaxation time of the fluid (e.g., see data in Figure 4b). Notably, all the aforementioned constraints that hinder the evaluation of the full spectrum of the fluid’s LVE properties are overcome by simply considering the motion of the particle in the azimuthal direction θ of the *Optical Halo*, where the particle is free to diffuse and thus capable to explore/reveal the full spectrum of the LVE properties of the suspending fluid. Note that the normalised position autocorrelation function for the azimuthal component is not reported in Figure 4b and d to maintain clarity in the diagrams. This is because the data would consistently hover near a value of unity throughout almost the entire time window, falling to zero only at large lag times. For the other components, these diagrams provide a valuable alternative to the particle’s mean squared displacement for determining the fluid’s LVE properties (because of Equation (12)).

**Figure 5:**
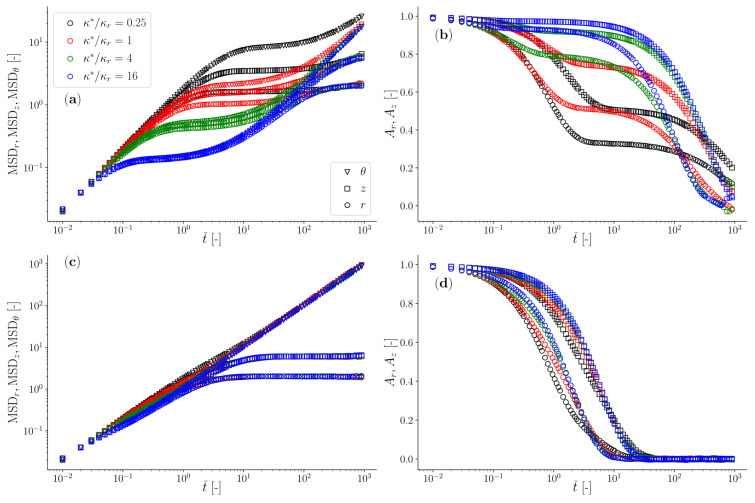
The mean squared displacement (**a**,**c**) and the normalised position autocorrelation function (**b**,**d**), evaluated along the three main directions *r*, *z* and θ of a toroidal optical trap, from Brownian dynamics simulations of an ensemble of 1000 particles on a torus with $\overset{ˇ}{R}=150$. The outcomes are parametric (colour coded) using the ratio of axial to radial stiffness $\kappa^{*}/\kappa_{r}$ as depicted in the legend. They show two cases: De=100 (**a**,**b**) and De=1 (**c**,**d**). Note that the normalised position autocorrelation function for the azimuthal component is not reported in order to maintain clarity in the diagrams. This is because the data would consistently hover near a value of unity throughout almost the entire time window, falling to zero only at large lag times.

We would like to share further results from our Brownian dynamics simulations, detailing how the dynamics of an optically trapped particle, suspended in a Jeffreys fluid and constrained by a toroidal optical trap, are influenced by variations in $\kappa^{*}$. Throughout these simulations, we maintain constant values for $\mathrm{De}=\tau /\zeta_{0}\kappa^{*}$ and $\kappa_{r}$. Consequently, changes in $\kappa^{*}$ lead to corresponding adjustments in τ, which exhibits an inverse relationship with $\kappa^{*}$ via De. In Figure 5, we demonstrate the temporal behaviour of both the particle’s MSD and the normalised position autocorrelation function for two cases: De=100 (top row) and De=1 (bottom row). Despite the fact that these results may appear to be repetitive with those shown in Figure 4, they highlight once again the advantage of adopting the *Optical halo* for those cases where the elastic component of the fluids is much weaker then the trap stiffness and has a significantly long relaxation time. This is evident for the case of De=100 and $\kappa^{*}/\kappa_{r}=0.25$ shown in Figure 5 (top row), for which the determination of the full frequency spectrum of the complex fluid is only hindered by the ‘patience’ of the operator to perform sufficiently long measurements so that the MSD may reach the linear regime, i.e., MSD∝t. For completeness of the discussion, we could summarise that all the cases where either $\kappa^{*}/\kappa_{r}>>1$ or De→1 do not represent a problem for classical single point optical tweezers, for which the reliability of the outputs would depend exclusively on the duration of the measurement whereas for all the other cases where $\kappa^{*}/\kappa_{r}<<1$ or De>>1, the only possible option for conducting broadband microrheology of complex fluids is to continuously monitor the azimuthal component of the particle trajectory. This requirement is further elucidated below by using a biologically relevant system as an example.

### 3.2. Simulation of a Biologically Relevant System with Realistic Numbers

**Figure 6:**
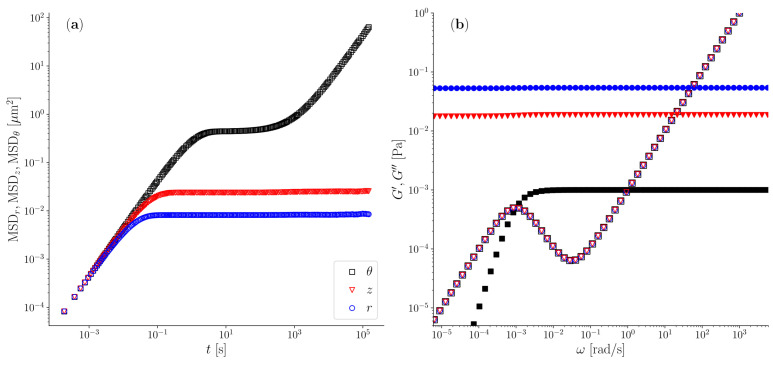
(**a**) The MSD along the three main directions *r*, *z*, and θ. (**b**) The viscoelastic moduli $G^{\prime }\left(\omega \right)$ and $G^{\prime \prime }\left(\omega \right)$ derived from each of the three MSD functions shown on the left. The MSD data have been obtained from Brownian dynamics simulations of an ensemble of 1000 particles on a torus with $\overset{ˇ}{R}=150$ and by assuming a value of the fluid’s plateau modulus of G=0.001 Pa, a relaxation time of τ=1000 s, and the solvent to have a viscosity of 0.001 Pa·s (e.g., water).

The enhanced optical tweezers configuration proposed in this study would enable operators to conduct wideband microrheology measurements on valuable biological systems, such as solutions of semiflexible biopolymers like reconstituted actin filaments (F-actin) for which a comprehensive characterisation of their mechanical spectrum, spanning from terminal to glassy regions, is still absent in the literature. Indeed, despite their significance in soft-matter physics, biology, and industrial processing, consensus on a fundamental analytical model for predicting the viscoelastic properties of semiflexible polymer solutions remains elusive. This is due to the diverse viscoelastic behaviours exhibited by solutions of semiflexible polymers, which vary depending on factors such as the polymers’ rigidity (often quantified by persistence length, $L_{p}$), their contour length (*L*), and the concentration (*c*) [35]. In the case of actin filament solutions, these typically exhibit a low frequency plateau modulus *G* that may range between 0.001 to 1 Pa and a relaxation times τ that can easily exceed 1000 s [30,31,32,33,34,35]. Therefore, if we assume a value of the plateau modulus of G=0.001 Pa, this yields $\kappa^{*}≃1.88\cdot 10^{-8}$ N/m, resulting in a ratio $\kappa^{*}/\kappa_{r}≃1.88\cdot 10^{-2}$, when the trap stiffness is assumed to be $\kappa_{r}=10^{-6}$. Moreover, by assuming a relaxation time of τ=1000 s and assuming the solvent to have a viscosity of 0.001 Pa·s (e.g., water), one would obtain a Deborah number of De=1000 which, together with a ratio of $\kappa^{*}/\kappa_{r}≃1.88\cdot 10^{-2}$, places these systems as excellent candidate to be investigated by means of the *Optical Halo*. In fact, the analysis of the azimuthal component of an optically trapped particle would be the ‘only’ microrheology approach capable of accessing the full frequency spectrum of the complex fluid, as depicted in Figure 6a, where the outcomes of Brownian dynamics simulations of a particle suspended in a Jeffreys fluid resembling a solution of actin filaments are presented in terms of the particle’s mean squared displacement for the main three cylindrical components [*r*,*z*,θ]. Notably, only the azimuthal component of the MSD is capable of revealing the low-frequency behaviour of the complex fluid, as elucidated in Figure 6b, where both the viscoelastic moduli of the fluid are correctly reported down to the terminal region, whereas the MSD curves of the other two components are governed by the compliance of the optical trap in the respective directions, which overshadow the fluid compliance at long times (here ∀t>0.1 s). This translates into an almost constant elastic modulus value for both the components [*r*,*z*], which are equal to $\kappa_{r}/\left(6\pi a\right)$ and $\kappa_{z}/\left(6\pi a\right)$, respectively, as shown in Figure 6b.

It follows that by conducting a sufficiently long microrheology measurement (e.g., ∼24 h) by means of a “*Haloed*” optical tweezers setup equipped with a high-acquisition-rate particle position detector (e.g., ∼0.1 MHz), one would be able to explore an unprecedented frequency range spanning over ten decades, from $10^{-5}$ to $10^{5}$ rad/s.

## 4. Conclusions

In conclusion, the *Optical Halo* microrheology technique is a novel and versatile approach for studying the viscoelastic properties of complex fluids, particularly for those systems characterised by a very long relaxation time. By harnessing the unique properties of ring-shaped Bessel beams generated by optical tweezers, we have shown through rigorous theoretical analysis and numerical simulations that the *Optical Halo* enables exploration of slow dynamics inaccessible to existing microrheology techniques. This opens up new avenues for investigating biological processes at micron scales with unprecedented precision and sensitivity. The *Optical Halo* technique represents a significant advancement in microrheology and has the potential to impact a wide range of scientific disciplines, from biophysics to materials science. Further experimental validation and application of this technique will undoubtedly contribute to our understanding of complex fluid behaviour and its implications in various fields of research.

## Data Availability

The original contributions presented in the study are included in the article, further inquiries can be directed to the corresponding author.

## Supplementary Materials

The following supporting information can be downloaded at: https://www.mdpi.com/article/10.3390/mi15070889/s1.
