# Supplementary material for: Optical Halo: A Proof of Concept for a New Broadband Microrheology Tool
Source: Micromachines (Basel). 2024 Jul 7;15(7):889. doi: 10.3390/mi15070889 (PMC11278636; doi:10.3390/mi15070889)
Supplement: Supplementary file 1 [file micromachines-15-00889-s001.zip › micromachines-3054397-supplementary.pdf]

micromachines

MDPI

Article

Supplementary Information "Optical Halo: A Proof of Concept for a New Broadband Microrheology Tool"

Jorge Ramírez [1], Graham M. Gibson [2] and Manlio Tassieri [3,*]

[1] Departamento de Ingeniería Química, Universidad Politécnica de Madrid, José Gutiérrez Abascal 2, 28006 Madrid, Spain; jorge.ramirez@upm.es
[2] School of Physics and Astronomy, Advanced Research Centre, University of Glasgow, Glasgow G11 6EW, UK; graham.gibson@glasgow.ac.uk
[3] Division of Biomedical Engineering, James Watt School of Engineering, Advanced Research Centre, University of Glasgow, Glasgow G11 6EW, UK
* Correspondence: manlio.tassieri@glasgow.ac.uk

Abstract: Microrheology, the study of material flow at micron scales, has advanced significantly since Robert Brown's discovery of Brownian motion in 1827. Mason and Weitz's seminal work in 1995 established the foundation for microrheology techniques, enabling the measurement of viscoelastic properties of complex fluids using light-scattering particles. However, existing techniques face limitations in exploring very slow dynamics, crucial for understanding biological systems. Here, we present a proof of concept for a novel microrheology technique called "*Optical Halo*", which utilises a ring-shaped Bessel beam created by optical tweezers to overcome existing limitations. Through numerical simulations and theoretical analysis, we demonstrate the efficacy of the *Optical Halo* in probing viscoelastic properties across a wide frequency range, including low-frequency regimes inaccessible to conventional methods. This innovative approach holds promise for elucidating the mechanical behaviour of complex biological fluids.

Keywords: rheology; microrheology; optical tweezers; complex fluids

check for updates

Citation: Ramírez, J. ; Gibson, G.M.; Tassieri, M. Optical Halo: A Proof of Concept for a New Broadband Microrheology Tool. *Micromachines* **2024**, *15*, 889. https://doi.org/10.3390/mi15070889

Academic Editors: Suvranta Tripathy and Yonggun Jun

Received: 27 May 2024
Revised: 4 July 2024
Accepted: 5 July 2024
Published: 7 July 2024

Copyright: © 2024 by the authors. Licensee MDPI, Basel, Switzerland. This article is an open access article distributed under the terms and conditions of the Creative Commons Attribution (CC BY) license (https://creativecommons.org/licenses/by/4.0/).

1. Jeffreys model and solution

Here, we provide the derivation of the constitutive equation and extract the complex modulus. Some of the derivations are standard in any book on viscoelasticity, but are included for the sake of completeness.

1.1. Version A

Considering the mechanical model depicted in Figure 1a, we have the following relationships:

$$\begin{aligned} \sigma_1 &= \sigma \\ \sigma_2 + \sigma_3 &= \sigma \\ \gamma_1 + \gamma_2 &= \gamma \end{aligned} \tag{S1}$$

as well as the following constitutive equations for the individual elements:

$$\begin{aligned} \sigma_1 &= \eta_1 \frac{d\gamma_1}{dt} \\ \sigma_2 &= G\gamma_2 \\ \sigma_3 &= \eta_3 \frac{d\gamma_3}{dt} \end{aligned} \tag{S2}$$

We start from the well-known constitutive relation for the Kelvin element (the spring and dashpot in parallel):

$$\sigma = \sigma_2 + \sigma_3 = G\gamma_2 + \eta_2 \frac{d\gamma_2}{dt} \tag{S3}$$

We now apply the same operator to the total deformation:

$$G\gamma + \eta_2 \frac{d\gamma}{dt} = G\gamma_1 + \eta_2 \frac{d\gamma_1}{dt} + G\gamma_2 + \eta_2 \frac{d\gamma_2}{dt} = G\gamma_1 + \eta_2 \frac{d\gamma_1}{dt} + \sigma \tag{S4}$$

Taking the time derivative of the previous expression, we get:

$$\begin{aligned} G\dot{\gamma} + \eta_2 \ddot{\gamma} &= G\dot{\gamma}_1 + \left(\frac{\eta_2}{\eta_1} + 1\right)\dot{\sigma} = G\frac{\sigma}{\eta_1} + \left(\frac{\eta_2}{\eta_1} + 1\right)\dot{\sigma} \\ G\dot{\gamma} + \eta_2 \ddot{\gamma} &= G\frac{\sigma}{\eta_1} + \frac{\eta_2 + \eta_1}{\eta_1}\dot{\sigma} \end{aligned} \tag{S5}$$

This can be further simplified to the obtain the final expression for the constitutive equation of the Jeffrey's model:

$$\eta_1 \dot{\gamma} + \frac{\eta_1 \eta_2}{G}\ddot{\gamma} = \sigma + \frac{\eta_2 + \eta_1}{G}\dot{\sigma} \tag{S6}$$

1.2. Version B

This version of the model is more intuitive, as it represents the well known Maxwell element (a spring and dashpot in series) in parallel with a viscous element. The model can be used to represent the viscoelastic behaviour of a diluted polymer solution, in which the polymer provides the viscoelastic behaviour and the solvent provides the background viscosity. In fact, this version of Jeffrey's model is directly related to the constitutive equation of Oldroyd B, frequently used in computational rheology. We first establish the relation for the Maxwell element:

$$\sigma_2 + \frac{\eta_2}{G}\dot{\sigma}_2 = \eta_2 \dot{\gamma} \tag{S7}$$

Knowing that the total stress $\sigma = \sigma_1 + \sigma_2$, we can apply the previous operator to the total stress:

$$\sigma + \frac{\eta_2}{G}\dot{\sigma} = \sigma_1 + \frac{\eta_2}{G}\dot{\sigma}_1 + \sigma_2 + \frac{\eta_2}{G}\dot{\sigma}_2 = \eta_1 \dot{\gamma} + \frac{\eta_2 \eta_1}{G}\ddot{\gamma} + \eta_2 \dot{\gamma} \tag{S8}$$

After some simplification, we get:

$$\sigma + \frac{\eta_2}{G}\dot{\sigma} = (\eta_1 + \eta_2)\dot{\gamma} + \frac{\eta_2 \eta_1}{G}\ddot{\gamma} \tag{S9}$$

The constitutive equations for the version A – eq. (S6) – and B – (S9) – are qualitatively very similar. In fact, it can be shown that if we choose:

$$\begin{aligned} \eta_1 &= \eta_1^* + \eta_2^* \\ \frac{\eta_1 \eta_2}{G} &= \frac{\eta_1^* \eta_2^*}{G^*} \\ \frac{\eta_2 + \eta_1}{G} &= \frac{\eta_2^*}{G^*} \end{aligned} \tag{S10}$$

the viscoelastic response of both versions of the Jeffreys model is exactly the same.

Complex modulus

The complex modulus can be directly extracted from the constitutive equation by using the Fourier transform. For example, Fourier transforming eq. (S6), we get:

$$\begin{aligned} \eta_1 i\omega\hat{\gamma} - \omega^2 \frac{\eta_1\eta_2}{G}\hat{\gamma} &= \hat{\sigma} + i\omega\frac{\eta_2+\eta_1}{G}\hat{\sigma} \\ \left(\eta_1 i\omega - \omega^2\frac{\eta_1\eta_2}{G}\right)\hat{\gamma} &= \left(1 + i\omega\frac{\eta_2+\eta_1}{G}\right)\hat{\sigma} \end{aligned} \tag{S11}$$

The complex modulus can be obtained as the ratio of the Fourier transform of the stress and the strain:

$$\begin{aligned} G^*(\omega) &= \frac{\hat{\sigma}}{\hat{\gamma}} = \frac{\eta_1 i\omega - \omega^2\frac{\eta_1\eta_2}{G}}{1 + i\omega\frac{\eta_2+\eta_1}{G}} \\ \frac{G^*(\omega)}{G} &= \frac{1}{G}\frac{\hat{\sigma}}{\hat{\gamma}} = \frac{i\omega\tau_1 - \omega^2\tau_1\tau_2}{1 + i\omega(\tau_2+\tau_1)} \end{aligned} \tag{S12}$$

where $\tau_1 = \eta_1/G$ and $\tau_2 = \eta_2/G$. By multiplying both the numerator and denominator by the complex conjugate of the denominator and doing some simplification, we can obtain an expression of the complex modulus of version A of the Jeffrey's model in which the storage and loss moduli are separated explicitly:

$$\frac{G^*(\omega)}{G} = \frac{1}{G}\frac{\hat{\sigma}}{\hat{\gamma}} = \frac{\omega^2\tau_1^2}{\left(1+\omega^2(\tau_2+\tau_1)^2\right)} + i\omega\tau_1\frac{1+\omega^2\tau_2(\tau_2+\tau_1)}{\left(1+\omega^2(\tau_2+\tau_1)^2\right)} \tag{S13}$$

Proceeding in a similar way with version B of the model, eq. (S9), we can extract the following expression for the complex modulus:

$$\frac{G^*(\omega)}{G} = \frac{1}{G}\frac{\hat{\sigma}}{\hat{\gamma}} = \frac{\omega^2\tau_2^2}{1+\omega^2\tau_2^2} + i\omega\left\{\tau_1 + \frac{\tau_2}{1+\omega^2\tau_2^2}\right\} \tag{S14}$$

For the right choice of parameters, both versions (a) and (b), the complex modulus and the viscosity of both versions of the model are identical, as can be seen in Figure S1.

Generalized Langevin equation for a particle moving in a Jeffrey's medium with optical tweezers

When a particle of mass m, trapped with optical tweezers, is diffusing through a viscoelastic medium, its motion is constrained by both the fluid and the elasticity of the trap. A mechanical model that represents the described situation, with a medium that behaves as a Jeoffrey's model, is given in Figure S2, where G_1 is the elastic constant of the optical trap. All three elements in parallel, carry a stress σ_i, with $i = 1 \ldots 3$, and the total stress is just $\sigma = \sigma_1 + \sigma_2 + \sigma_3$. In order to extract the constitutive equation, we first establish the relation for the Maxwell element (a spring and a dashpot in series):

$$\sigma_2 + \frac{\eta_2}{G_2}\dot{\sigma}_2 = \eta_2\dot{\gamma} \tag{S15}$$

If we apply the same operator to the total stress, we get:

$$\begin{aligned} \sigma + \frac{\eta_2}{G_2}\dot{\sigma} &= \sigma_1 + \frac{\eta_2}{G_2}\dot{\sigma}_1 + \sigma_2 + \frac{\eta_2}{G_2}\dot{\sigma}_2 + \sigma_3 + \frac{\eta_2}{G_2}\dot{\sigma}_3 \\ &= G_1\gamma + \frac{\eta_2}{G_2}G_1\dot{\gamma} + \eta_2\dot{\gamma} + \eta_3\dot{\gamma} + \frac{\eta_2\eta_3}{G_2}\ddot{\gamma} \\ &= G_1\gamma + \left(\left(1+\frac{G_1}{G_2}\right)\eta_2 + \eta_3\right)\dot{\gamma} + \frac{\eta_2\eta_3}{G_2}\ddot{\gamma} \end{aligned} \tag{S16}$$

We can Fourier transform the constitutive equation, to get:

$$\hat{\sigma}\left(1+i\omega\frac{\eta_2}{G_2}\right)=\hat{\gamma}\left[G_1+i\omega\left(\left(1+\frac{G_1}{G_2}\right)\eta_2+\eta_3\right)-\omega^2\frac{\eta_2\eta_3}{G_2}\right] \tag{S17}$$

From the constitutive equation in Fourier space, we can extract the complex modulus:

$$G^*(\omega)=\frac{\hat{\sigma}(\omega)}{\hat{\gamma}(\omega)}=\frac{\left[G_1+i\omega\left(\left(1+\frac{G_1}{G_2}\right)\eta_2+\eta_3\right)-\omega^2\frac{\eta_2\eta_3}{G_2}\right]}{\left(1+i\omega\frac{\eta_2}{G_2}\right)} \tag{S18}$$

which can be simplified to:

$$G^*(\omega)=G_1+G_2\frac{\omega^2\tau_2^2}{(1+\omega^2\tau_2^2)}+i\omega\left[G_1\tau_3+\frac{G_2\tau_2}{(1+\omega^2\tau_2^2)}\right] \tag{S19}$$

The compliance and the complex modulus are related by the following expression:

$$|G^*(\omega)|=\frac{1}{|J^*(\omega)|} \tag{S20}$$

And thus, the modulus of the complex compliance takes the form:

$$|J^*(\omega)|=\frac{\left(1+\omega^2\tau_2^2\right)}{G_1\omega^2\tau_2^2}\frac{1}{\sqrt{\frac{1}{\omega^4\tau_2^4}+\frac{2}{\omega^2\tau_2^2}\left[1+\frac{G_2}{G_1}+\frac{1}{2}\left(\frac{\tau_3}{\tau_2}+\frac{G_2}{G_1}\right)^2\right]+\left[\left(1+\frac{G_2}{G_1}\right)^2+2\frac{\tau_3^2}{\tau_2^2}\left(1+\frac{G_2}{G_1}\frac{\tau_2}{\tau_3}\right)\right]+\omega^2\tau_3^2}} \tag{S21}$$

By substituting the frequency ω by $1/\tau$, we can get the compliance in the time domain:

$$|J^*(1/\tau)|=\frac{\left(\tau^2+\tau_2^2\right)}{\tau_2^2G_1}\frac{1}{\sqrt{\frac{\tau^4}{\tau_2^4}+2\frac{\tau^2}{\tau_2^2}\left[1+\frac{G_2}{G_1}+\frac{1}{2}\left(\frac{\tau_3}{\tau_2}+\frac{G_2}{G_1}\right)^2\right]+\left[\left(1+\frac{G_2}{G_1}\right)^2+2\frac{\tau_3^2}{\tau_2^2}\left(1+\frac{G_2}{G_1}\frac{\tau_2}{\tau_3}\right)\right]+\frac{\tau_3^2}{\tau^2}}} \tag{S22}$$

which allows us to extract the two asymptotic limits as:

$$\lim_{\tau\to 0}|J^*(1/\tau)|\cong\frac{1}{G_1}\frac{\tau}{\tau_3}=\frac{\tau}{\eta_3} \tag{S23}$$

and

$$\lim_{\tau\to\infty}|J^*(1/\tau)|\cong\frac{\left(\tau^2+\tau_2^2\right)}{\tau^2G_1}\cong\frac{1}{G_1} \tag{S24}$$

A characteristic representation of the shape of $|J^*(1/\tau)|$ is shown in Figure S3.

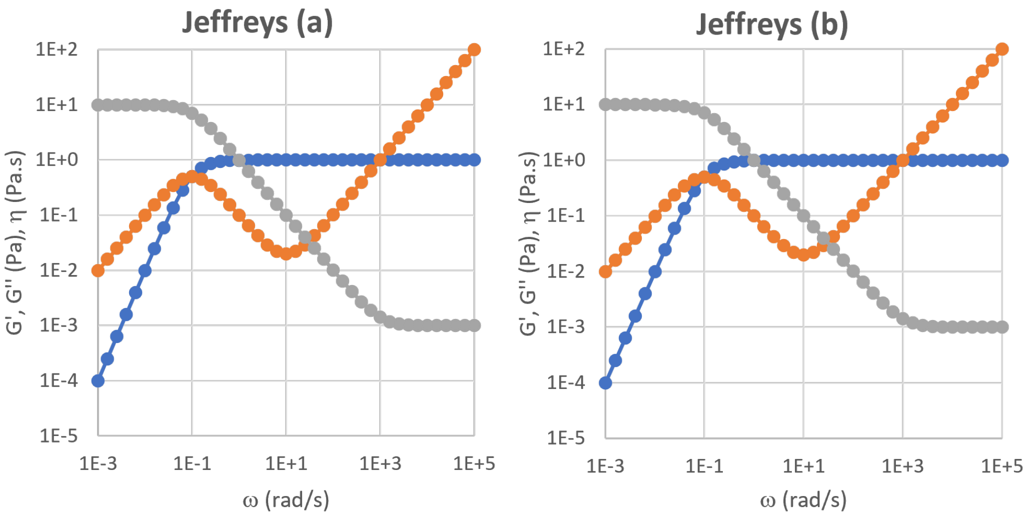

Figure S1. Storage modulus (blue), loss modulus (orange) and viscosity (grey) of versions (a) and (b) of the Jeffreys model. For version (a), $\tau_1 = 10$ and $\tau_2 = 10^{-3}$. For version (b), $\tau_1 = 10^{-3}$ and $\tau_2 = 10$.

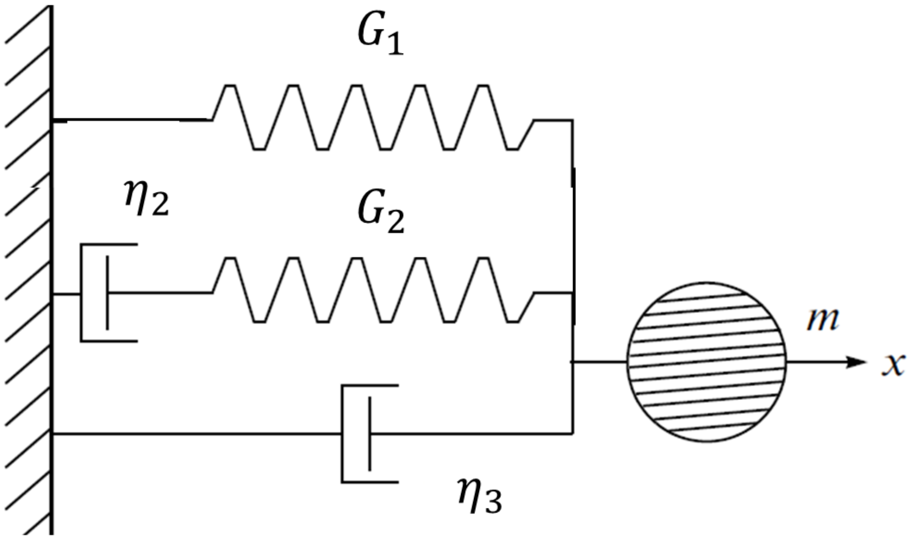

Figure S2. Mechanical model for a particle moving in a Jeffreys medium and trapped by optical tweezers.

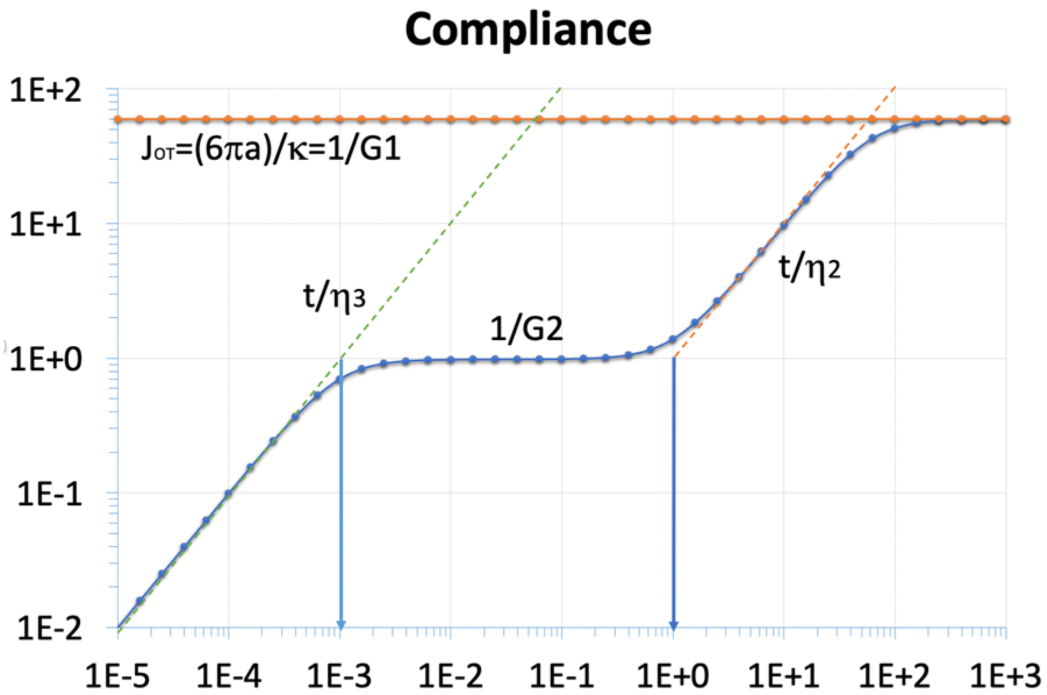

Figure S3. Compliance of a particle moving in a Jeffreys medium and trapped by optical tweezers.

Disclaimer/Publisher's Note: The statements, opinions and data contained in all publications are solely those of the individual author(s) and contributor(s) and not of MDPI and/or the editor(s). MDPI and/or the editor(s) disclaim responsibility for any injury to people or property resulting from any ideas, methods, instructions or products referred to in the content.
